# The Pro-Fibrotic Response to Lens Injury Is Signaled in a PI3K Isoform-Specific Manner

**DOI:** 10.3390/biom12091181

**Published:** 2022-08-25

**Authors:** A. Sue Menko, Janice L. Walker

**Affiliations:** 1 Department of Pathology, Anatomy and Cell Biology, Sidney Kimmel Medical College, Thomas Jefferson University, Philadelphia, PA 19107, USA; 2 Department of Ophthalmology, Sidney Kimmel Medical College, Thomas Jefferson University, Philadelphia, PA 19107, USA

**Keywords:** PI3K, PI3Kp110α, fibrosis, myofibroblast, proliferation, lens

## Abstract

The signaling inputs that function to integrate biochemical and mechanical cues from the extracellular environment to alter the wound-repair outcome to a fibrotic response remain poorly understood. Here, using a clinically relevant post-cataract surgery wound healing/fibrosis model, we investigated the role of Phosphoinositide-3-kinase (PI3K) class I isoforms as potential signaling integrators to promote the proliferation, emergence and persistence of collagen I-producing alpha smooth muscle actin (αSMA+) myofibroblasts that cause organ fibrosis. Using PI3K isoform specific small molecule inhibitors, our studies revealed a requisite role for PI3K p110α in signaling the CD44+ mesenchymal leader cell population that we previously identified as resident immune cells to produce and organize a fibronectin-EDA rich provisional matrix and transition to collagen I-producing αSMA+ myofibroblasts. While the PI3K effector Akt was alone insufficient to regulate myofibroblast differentiation, our studies revealed a role for Rac, another potential PI3K effector, in this process. Our studies further uncovered a critical role for PI3K p110α in signaling the proliferation of CD44+ leader cells, which is important to the emergence and expansion of myofibroblasts. Thus, these studies identify activation of PI3K p110α as a critical signaling input following wounding to the development and progression of fibrotic disease.

## 1. Introduction

Matrix proteins, growth factors and cytokines, all important factors in the reparative response to injury, are also linked to the induction of fibrosis [1,2,3,4,5,6,7]. Whether the wound-repair outcome they induce is reparative or fibrotic can be dependent on coordination in their activation of downstream effector signaling pathways [8,9,10]. A key cellular target for these factors is the mesenchymal cells that respond to injury, primarily the fibroblasts and immune cells that rapidly populate the wound site [4,11,12,13,14]. In reparative healing, including the response to cataract surgery wounding, mesenchymal cells guide wound closure [11,15,16,17]. However, the matrix and soluble factors induced in the repair environment can instead induce these mesenchymal cell populations to acquire a myofibroblast phenotype [18], which in the post-cataract surgery lens is responsible for the fibrotic lens disease known as Posterior Capsule Opacification (PCO) [3,19,20]. The expression of αSMA by these mesenchymal cells, its assembly into contractile stress fibers, and their production of collagen I, underlie the development of fibrosis post-wounding [21].

The initial appearance of myofibroblasts following wounding is often to promote the reparative process. These myofibroblasts do not persist after the wound is healed. Instead, they become senescent, and are typically programmed to undergo apoptotic cell death [22]. However, when this program is countered by persistent signals in the environment that promote their proliferation and survival, they populate the wound site in large numbers and create the collagen I-rich environment that destroys tissues in fibrosis [22]. Among the primary signal transducers of both cell proliferation and survival are Phosphatidylinositol 3-kinase (PI3K)-dependent signaling pathways [23,24]. The Class I PI3Ks are comprised of catalytic (p110α, β, δ, and γ) and regulatory (p85) subunits [24]. These different PI3K p110 subunits can be activated downstream of many upstream inducers, primarily integrins, growth factor receptors and/or G-protein coupled receptors [24,25,26], and in turn lead to the activation of many downstream effector pathways including Akt and Rac [24]. As such, the PI3Ks have been linked to many different functions central to development, tissue homeostasis and pathogenesis, with distinct roles in each depending on the context. Often the full complexity of the multitude of PI3K responses, including the identification of roles for different isoforms and downstream effector pathways remains unknown. In the response to wounding, PI3K has been linked to inducing the transition of mesenchymal cells to a myofibroblast fate and to the development of fibrosis when activated by growth factors, including TGFβ1 and PDGF-BB, and integrin family members like the fibronectin receptor α5β1 [27,28]. In determining whether wound repair is regenerative or fibrotic, the specific roles of PI3Ks also appear to be context specific, their pro-fibrotic signaling function exemplified in studies of idiopathic pulmonary fibrosis in which the transition of lung fibroblasts to myofibroblasts has been linked to activation of PI3K signaling downstream of TGFβ1 [28,29]. Much remains to understand about how specific PI3K p110 isoforms and their effector pathways regulate both the emergence and persistence of myofibroblasts to establish a fibrotic wound-repair outcome.

In studies with ex vivo mock cataract surgery explant cultures that mimic the in vivo fibrotic outcome of cataract surgery known as PCO, our studies show that the wound edge is rapidly populated by lens resident immune cells that guide the migration of the wounded lens epithelium [16,30]. The expression of TGFβ in these ex vivo post-cataract surgery wounded explants induces the lens resident immune cells at the wound edge to produce a fibronectin-EDA-rich matrix environment in which these mesenchymal cells acquire a myofibroblasts phenotype [3]. In this environment, the αSMA+ myofibroblasts continue to expand in number and express and organize a collagen I-rich matrix [3,16,31].

Much remains to understand about the signaling pathways that promote the persistence of myofibroblasts following a wounding and the development of fibrosis. Our studies show a specific link between activation of the PI3K p110α subunit and the appearance and proliferation of myofibroblasts following a wounding.

## 2. Materials and Methods

### 2.1. Ex vivo Post-Cataract Surgery Chick Explant Cultures and Inhibitor Treatments

Ex vivo post-cataract surgery chicken explant lenses were prepared as previously described [17,32]. Please refer to [16,33,34,35] for schematic depiction of this wound healing/fibrosis model. Mock cataract surgery was performed on lenses isolated from E14/E15 chick embryos. Mock cataract surgery removes the lens fiber cells, which make up the bulk of the lens, leaving behind the lens epithelial cells that are tightly adherent to the lens capsule, the basement membrane structure that surrounds the lens. Cuts are introduced to the lens epithelium to flatten the culture cell side up on the tissue culture substrate resulting in the wounded lens explant. Eggs were obtained from Poultry Futures (Lititz, PA, USA). All animal studies are approved by the Institutional Animal Care and Use Committee (IACUC) at Thomas Jefferson University (Philadelphia, PA, USA). For studies with both pan-Phosphoinositide 3-kinase (PI3K) and PI3K isoform specific inhibitors, ex vivo post-cataract surgery explants were treated from either day 0 (D0) or D1 through to D3 in culture with the pan PI3K inhibitor LY294002 (LY) at 25 µM (Selleckchem, Houston, TX, USA, the PI3Kα specific inhibitor HS-173 at 1 µM (Selleckchem, Houston, TX, USA), the PI3K p110β-specific inhibitor TGX-221 at 3 µM (Selleckchem, Houston, TX, USA, or the PI3Kδ/γ inhibitor Duvelisib (IPI-145,INK-197) at 3 µM (Selleckchem, Houston, TX, USA). To determine if PI3K mediates fibrosis through its effectors AKT and/or Rac, ex vivo post-cataract surgery explant cultures were treated with the allosteric Akt inhibitor MK-2206 at 1 µM and 3 µM (Selleckchem, Houston, TX, USA, or the Rac inhibitors; NSC23766 at 20 µM or EHop-016 at 1 µM. Both vehicle (DMSO) and inhibitors were replaced each day, and at D3 post-injury cultures were either fixed in 4% formaldehyde or extracted in RIPA buffer (5 mM EDTA, 150 mM NaCl, 1% NP40, 1% sodium deoxycholate, 1% SDS 20% solution, 50 mM Tris-HCl, pH 7.4) with protease/phosphatase inhibitor cocktail (Cell Signaling Technology, Aldrich, Danvers, MA, USA) for Western blot/WES analysis. 

### 2.2. Immunofluorescence 

For immunolabeling studies, ex vivo post-cataract surgery explant cultures were fixed in 4% formaldehyde for 15 min. Subsequently, cultures were permeabilized in 0.25% Triton-X-100 for 10 min, blocked in 5% goat serum for 30 min, incubated with primary antibodies for 30 min to 1hr followed by incubation with fluorescent-conjugated secondary antibodies (Jackson ImmunoResearch, West Grove, PA, USA). For immunolabeling we used the following primary antibodies αSMA (Sigma Aldrich, St. Louis, MO or Abcam, Cambridge, MA, USA), Fibronectin EDA (Santa Cruz Biotechnology, Santa Cruz, CA, USA), active RAC (NewEast Biosciences, King of Prussia, PA, USA) and a vimentin polyclonal (Abcam, Cambridge, MA, USA). The following primary antibodies were obtained from the Developmental Studies Hybridoma Bank (DSHB) created by the NICHD of the NIH (The University of Iowa, Department of Biology, Iowa City, IA, USA): pro-collagen I antibody (SP1.D8) deposited to the DSHB by Furthmayr, H. (DSHB Hybridoma Product SP1.D8) and CD44 antibody (1D10) deposited to the DSHB by Halfter, W.M. (DSHB Hybridoma Product 1D10). Explant cultures were counterstained with DAPI (Biolegend, San Diego, CA, USA) to identify nuclei.

### 2.3. Proliferation

Proliferation was analyzed using an EdU click it assay kit (ThermoFisher Scientific, Waltham, MA, USA). These post-cataract surgery cultures then were immunolabeled with CD44 to identify resident immune cells at the leading edge and nuclei were co-stained with DAPI. Labeled cultures were examined by confocal microscopy.

### 2.4. TUNEL Assay

Apoptosis was analyzed in the post-cataract surgery explant cultures using a TUNEL assay kit (Sigma Aldrich, St. Louis, MO, USA) that detects double strand DNA breaks, which were co-stained with DAPI and examined by confocal microscopy.

### 2.5. Immunoblotting

Western Blot: This immunoblot analysis was performed as described previously [35]. Briefly, post-cataract surgery wounded explant cultures were extracted in RIPA, and 10–20 μg protein was loaded onto an SDS-PAGE 4–16% tris/glycine gels (Invitrogen, Waltham, MA, USA). Proteins were electrophoretically transferred from the gels onto Immobilon-P membranes (Millipore, Burlington, MA, USA). Subsequently, membranes were blocked in 5% milk, probed for primary antibodies at 4 °C overnight and exposed to secondary antibodies conjugated to horseradish peroxidase (HRP) (Bio-Rad, Hercules, CA, USA) for 1 h at room temperature. Bands were detected after incubation with the ECL reagent (Thermo Fisher Scientific) using the FluorChem E & M Imager (ProteinSimple, San Jose, CA, USA). For immunoblotting we used the following primary antibodies: αSMA (Sigma Aldrich, St. Louis, MO, USA or Abcam, Cambridge, MA, USA), GAPDH (Santa Cruz Biotechnology, Santa Cruz, CA, USA), pAKT (Ser473) (Cell Signaling Technology, Danvers, MA, USA), total Akt (Cell Signaling Technology, Danvers, MA, USA), pGSK (Ser9) (Cell Signaling Technology, Danvers, MA, USA) and total GSK (MilliporeSigma, Burlington, MA, USA).

Simple Western system by Protein Simple (WES, New York, NY, USA): Post-cataract surgery wounded explant cultures were extracted in RIPA buffer. Samples were prepared for WES immunoblot as previously described [36]. Briefly, samples were separated using capillary electrophoresis to detect proteins based on their size using chemiluminescence. WES immunoblotting was performed with primary antibodies pAKT and total Akt.

### 2.6. Confocal Microscopy Imaging

Confocal imaging was performed using either the Zeiss 510 or Zeiss 800 confocal microscopes. Z-stacks were collected with a 40× objective, each optical plane at either 0.33 µm or 0.49 µm, as indicated. For z-stacks collected with a 20× objective each optical plan was at 0.6 µm. Images are shown as either single optical planes from the z-stack or as projected images, as indicated.

### 2.7. Statistical Analysis 

Data are shown as the mean +/− SEM and analyzed with unpaired Student’s t-test or by ANOVA using Prism (Graphpad Software Prism 9, La Jolla, CA, USA). *p*-value of 0.05 or less was considered statistically significant. Quantification of protein expression for all Western blots was performed using ProteinSimple analysis software. For WES analysis, signal intensity is obtained using COMPASS software to obtain signal intensity by quantifying the area under the curve of the acquired graph. The % of CD44+ cells that are EdU+ were calculated over 3 independent experiments.

## 3. Results

### 3.1. PI3K Signals Transition to a Fibrotic Phenotype in Response to a Mock Cataract Surgery Wounding

**Figure 1:**
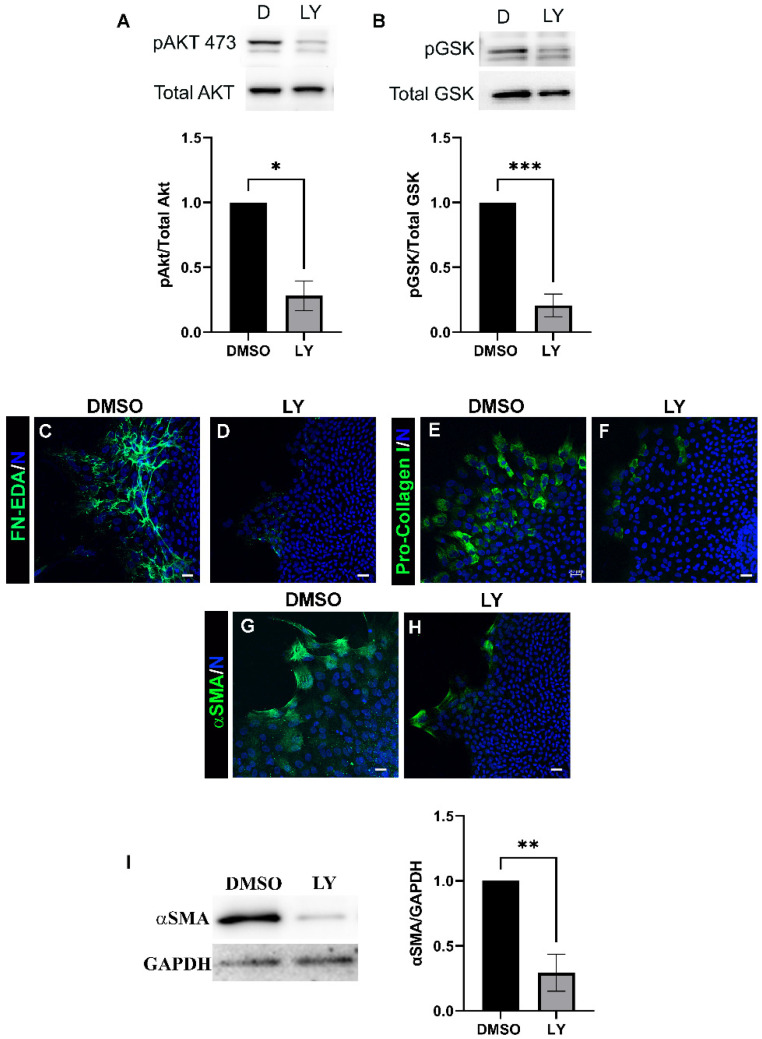
PI3K signals production of FN-EDA, pro-collagen I and differentiation into αSMA+ myofibroblasts post-wounding. Ex vivo post-cataract surgery explants were treated with vehicle (DMSO) or pan-PI3K inhibitor LY294002 (LY) on day 1 through day 3 and subjected to immunoblot analysis (**A**,**B**) to determine the activation state of the PI3K effector Akt and phosphorylation of GSK by immunoblotting (**A**) phosphoSer473-Akt (pAkt) and total Akt and for (**B**) phospho-GSK (Ser9) and total GSK. Results show that LY294402 treatment effectively suppressed Akt and GSK phosphorylation. Quantification of the relative intensity of pAkt/total Akt, pGSK/total GSK normalized to DMSO is displayed in bar graphs. (**C**–**H**) D3 LY294002 and vehicle treated ex vivo post-cataract surgery explant cultures were immunolabeled for (**C**,**D**) FN-EDA, (**E**,**F**) pro-Col I, (**G**,**H**) αSMA, co-labeled for DAPI and imaged by confocal microscopy. LY294002 treatment effective suppressed FN-EDA, pro-Col I and αSMA. All images are presented as projections from collected confocal z-stacks. Magnification bars = 20 µm. (**I**) Immunoblots of Day 3 LY294002 and vehicle treated ex vivo post-cataract surgery explants shown for αSMA and loading control GAPDH. Quantification of the relative intensity of αSMA and GAPDH normalized to DMSO is displayed in bar graphs (*n* = 3). Error bars represent S.E.M., * *p* ≤ 0.05, ** *p* ≤ 0.01, and *** *p* ≤ 0.001.

PI3K is a major downstream effector of multiple receptor inputs linked to driving fibrosis, such as TGFβ and integrins [37,38,39,40,41]. We examined the role of PI3Ks in a clinically relevant ex vivo post-cataract surgery chick explant wound healing/fibrosis model in which the resident immune cells activated to populate the wound edge and direct migration of the wounded lens epithelium across the culture substrate acquire a myofibroblast phenotype [30,35]. For these studies, the ex vivo wounded post-cataract surgery cultures were exposed to the pan PI3K inhibitor LY294002, which blocks all class I PI3K isoforms, or to its vehicle DMSO, from D1 through D3 post-injury at which time the CD44+ mesenchymal immune cell leader population first transitions to alpha smooth muscle actin (αSMA)+ myofibroblasts [35]. Their acquisition of a myofibroblast phenotype is preceded by their production of a Fibronectin-EDA (FN-EDA) provisional matrix on the substrate surface, and coincident with the production of a Collagen I (Col-I) matrix [3,35,42]. To determine the efficacy of LY294002 on inhibition of PI3K signaling in the ex vivo mock cataract surgery cultures we examined the activation state (phosphorylation) of its effector Akt (p-ser 473) and the phosphorylation of the Akt target Glycogen Synthase Kinase-3 at ser9 (GSK3) [43,44]. Treatment with LY294002 effectively suppressed activation of Akt and phosphorylation of GSK (Figure 1A,B). To determine the effect of blocking PI3K signaling with LY294002 on the development of fibrosis we performed confocal microscopy imaging following immunolabeling for FN-EDA (Figure 1C,D), pro-Col I (Figure 1E,F) or αSMA+ (Figure 1G,H). The results showed that exposure to LY294002 prevented the production of FN-EDA and Col I in the extracellular microenvironment of the leader cells and the emergence of αSMA+ myofibroblasts. Western blot analysis for αSMA+ also showed significant suppression of expression of this myofibroblast protein (Figure 1I). These studies show that PI3K signaling is required for the formation of a FN-EDA and collagen I rich matrix and induction of the myofibroblast phenotype in response to cataract surgery wounding that is necessary for the development of fibrosis.

### 3.2. Acquisition of an αSMA+ Myofibroblast Phenotype in Response to Lens Injury Is Induced in a PI3K-Isoform Specific Manner

**Figure 2:**
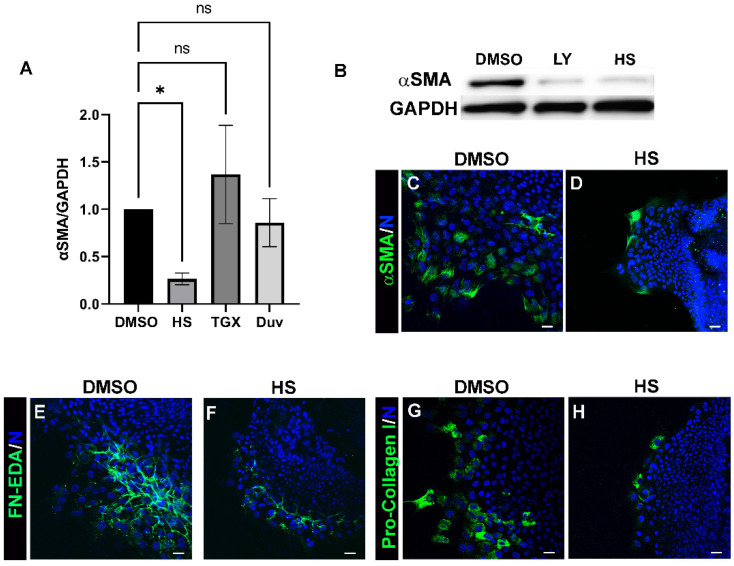
The fibrotic response of mesenchymal leader cells is signaled through PI3K 110α post-cataract surgery wounding. (**A**) Ex vivo post-cataract surgery explants were treated with vehicle or PI3K isoform specific inhibitors: HS-173 (HS), a PI3K p110α specific inhibitor; TGX-221 (TGX), a PI3K p110β-specific inhibitor; or Duvelisib (Duv) a PI3Kδ/γ selective inhibitor through day 3. Lysates were subjected to immunoblot analysis to determine the effect on αSMA expression. Quantification of the relative intensity of αSMA and GAPDH normalized to DMSO is displayed in the bar graph (*n* > 3). (**B**) Representative immunoblot showing the effect of treatment with the pan PI3K inhibitor LY294002 and the PI3K 110α specific inhibitor HS-173 vs. vehicle control on αSMA and GAPDH (loading control) protein expression D3 post-treatment. Both LY294002 and HS-173 show a similar effect on reducing αSMA expression levels without effecting GAPDH. These findings provide further evidence that myofibroblast differentiation is signaled largely through the PI3Kα isoform. (**C**–**H**) D3 HS-173 and vehicle treated ex vivo post-cataract surgery explant cultures were immunolabeled for (**C**,**D**) αSMA, (**E**,**F**) FN-EDA, and (**G**,**H**) pro-Col I, co-labeled for DAPI and imaged by confocal microscopy. HS treatment effectively suppressed αSMA+ myofibroblast emergence and the accumulation of FN-EDA and pro-Col I. All images are presented as a single optical plane from collected confocal z-stacks. Error bars represent S.E.M., * *p* ≤ 0.05, ns: not significant. Magnification bar = 20 µm.

Our findings show that class I PI3Ks serve as crucial intracellular signaling coordinators of the fibrotic response to lens cataract surgery wounding. The catalytic subunit of class I PI3K is found in four different isoforms, of which p110α, p110β, and p110γ have been shown to be expressed in the lens [36], and each of which may have distinct signaling functions in the response to lens injury. We investigated if the impact of PI3K signaling on induction of leader cell transition to an αSMA+ myofibroblast fate in response to cataract surgery wounding is PI3K isoform specific. For these studies, ex vivo wounded post mock cataract surgery explant cultures were exposed to vehicle DMSO or PI3K isoform specific inhibitors including HS-173 (HS), specific for p110α; TGX-221(TGX), specific for p110β; or Duvelisib (Duv) a selective inhibitor for p110δ/γ, from either D0 or D1 through to D3 post-injury and immunoblotted for αSMA (Figure 2A). Only inhibition of p110α paralleled the impact of the pan-PI3K inhibitor on suppressing αSMA expression following cataract surgery wounding. Inhibition of the transition of the resident immune leader cell population to αSMA+ myofibroblasts by blocking activation of PI3K p110α with HS-173 was shown by both immunoblot (Figure 2B) and immunolocalization analyses (Figure 2C,D). These findings demonstrate that activation of p110α is alone sufficient to activate a pro-fibrotic signaling pathway in response to cataract surgery wounding. To investigate this further, wounded ex vivo explants exposed to vehicle or the p110α specific inhibitor HS-173 were immunolabeled for FN-EDA (Figure 2E,F) or pro-collagen I (Figure 2G,H). Consistent with the inhibition of transition of the mesenchymal leader cells to an αSMA+ myofibroblast phenotype, inhibition of p110α suppressed the assembly of FN-EDA and production of pro-collagen I by these cells. These studies reveal that p110α is the principal PI3K isoform involved in signaling the induction of the fibrotic phenotype in response to lens wounding.

### 3.3. Blocking Akt Activation Is Alone Insufficient to Block Myofibroblast Emergence

**Figure 3:**
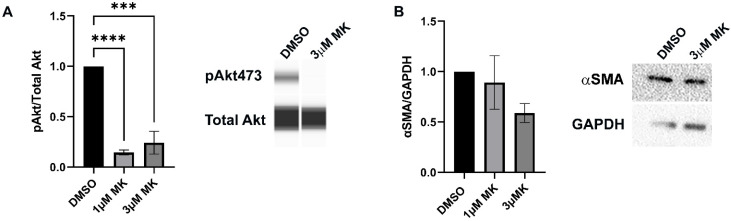
Role of Akt in mediating myofibroblast differentiation. (**A**,**B**) Ex vivo post-cataract surgery wounded explants were treated with vehicle (DMSO) or the Akt specific inhibitor MK-2206 (MK) on day 1 through day 3 and subjected to immunoblot analysis. (**A**) The activation state of the PI3K effector Akt was determined by immunoblotting for phosphoSer473-Akt (pAkt) and total Akt. Quantification of the relative intensity of pAkt/total Akt, normalized to DMSO is displayed in the bar graph. (**B**) Immunoblots of D3 vehicle and MK2206 treated ex vivo post-cataract surgery explant lysates shown for αSMA and loading control GAPDH. Quantification of the relative intensity of αSMA and GAPDH normalized to DMSO is displayed in bar graphs (*n* = 3). Results show that MK treatment effectively suppressed activation of Akt and that there was a dose dependent decrease in αSMA expression; however, the changes in αSMA expression were not found to be statistically significant. Error bars represent S.E.M., *** *p* ≤ 0.001, **** *p* ≤ 0.0001.

As Akt is a major downstream effector of PI3K, we examined whether its activation was responsible for inducing the resident immune cells at the leading wound edge to transition to an αSMA+ myofibroblast fate. Ex vivo post-cataract surgery wounded lens explant cultures were treated with vehicle or the Akt specific inhibitor MK-2206 at both 1 µM or 3 µM from D1 through D3 post-injury. Both inhibitor concentrations effectively suppressed activation of Akt (Figure 3A). While Western Blot analysis showed a dose-dependent trend of decreased αSMA expression when Akt activation was blocked, it was not statistically significant (Figure 3B). These findings suggest that the PI3K downstream effector Akt is not alone sufficient to signal the emergence of myofibroblasts associated with the fibrotic response to cataract surgery wounding.

### 3.4. Rac Signaling Is Involved in Promoting αSMA+ Myofibroblast Differentiation in Response to Cataract Surgery Wounding

**Figure 4:**
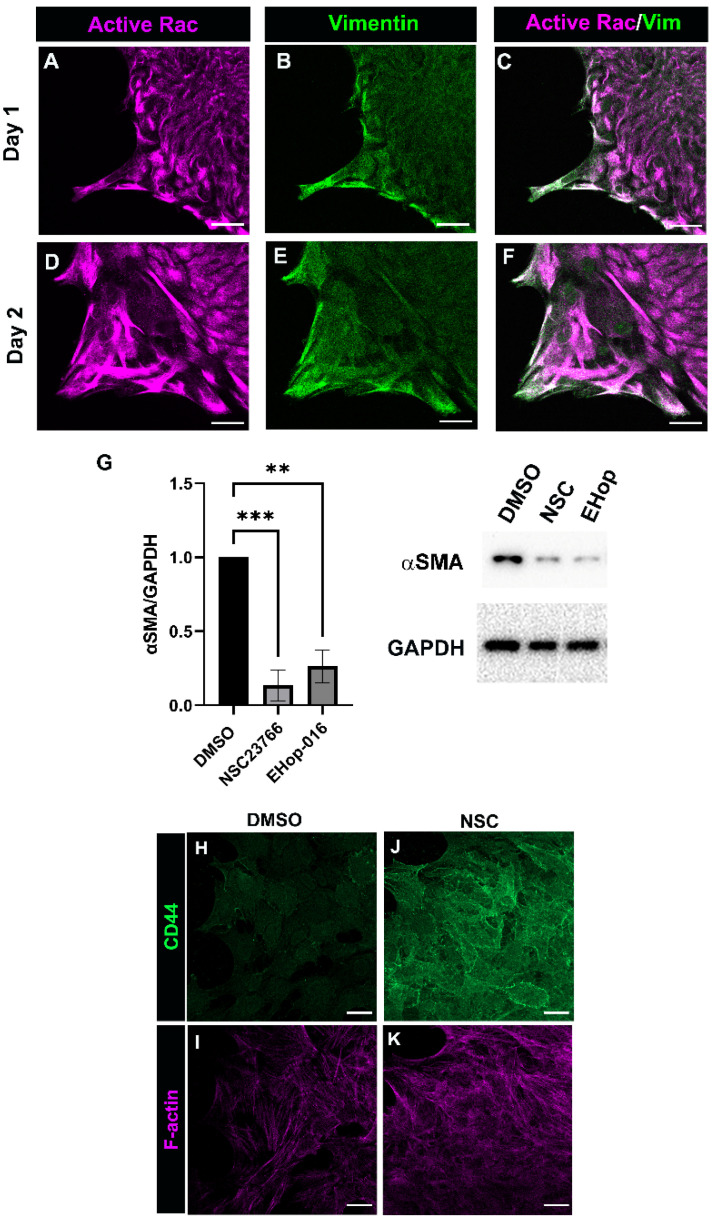
Rac signaling is involved in inducing myofibroblast differentiation. (**A**–**F**) Ex vivo post-cataract surgery explant cultures at (**A**–**C**) D1 and (**D**–**F**) D2 were immunolabeled for active Rac (**A**,**D**) and vimentin (**B**,**C**,**E**,**F**) and imaged at the leading edge by confocal microscopy. (**C**,**F**) Image overlays for Rac and vimentin show their co-localization to protrusions extended by leader cells. Images are presented as a single optical plane from the collected confocal z-stack with each optical plane at 0.49 µm. Magnification bar = 20 µm. (**G**) Ex vivo post-cataract surgery explants were treated with vehicle (DMSO) or Rac specific inhibitors NSC23766 (NSC) or EHop-016 (EHop) on day 1 through day 3 post-injury and subjected to immunoblot analysis for αSMA and the loading control GAPDH. Quantification of the relative intensity of αSMA and GAPDH normalized to DMSO is displayed in bar graphs (*n* = 3 for NSC23766, *n* = 2 for EHop-016). Results show that Rac signaling is involved in inducing αSMA expression. (**H**–**K**) Ex vivo post-cataract surgery explants cultured for 48hrs in the presence of either DMSO or NSC were labeled for CD44 (**H**,**J**) and F-actin (**I**,**K**) and imaged at the leading edge by confocal microscopy. Results show that CD44 expression was retained by leader cells culture in the presence of NSC. Images are presented as projected images from collected confocal z-stacks (**H**,**J**). Images for F-actin labeling are shown as a single optical plane from collected confocal z-stacks (**I**,**K**). Error bars represent S.E.M., ** *p* ≤ 0.01, and *** *p* ≤ 0.001.

Another major downstream effector of PI3K is the small GTPase Rac [45,46], a well-known regulator of cytoskeletal dynamics [47,48], which is also directly implicated in the regulation of fibrosis [49,50,51]. Post-cataract surgery explant cultures were co-immunolabeled at D1 and D2 post-wounding for active Rac (Figure 4A,C,D,F) and vimentin (Figure 4B,C,E,F), a cytoskeletal protein enriched in the protrusions that mesenchymal leader cells extend at the leading edge [35]. Our previous findings show that the vimentin function is required for the transition of the leader cells to a myofibroblast phenotype [31,35]. Confocal microscopy imaging showed that active Rac was co-localized with vimentin in the lamellipodia protrusions of mesenchymal leader cells prior to their transition to myofibroblasts. These findings reveal a potential Rac/vimentin coordinated function in leader cell protrusions and poise Rac as a regulator of leader cell function in response to cataract surgery wounding.

To determine whether Rac is involved in mediating the transition of the leader cells to αSMA+ myofibroblast phenotype, ex vivo post cataract surgery lens wounded explant cultures were treated from D1 through D3 with vehicle (DMSO) or with the Rac 1 inhibitors NSC23766 or EHop-016. Western blot analysis showed that the inhibition of Rac activation using either NSC23766 or EHop-016 resulted in a significant suppression of αSMA expression (Figure 4G). Consistent with finding that CD44 expression is suppressed in leader cells as they become myofibroblasts [3], we now show that CD44 is retained by the leader cells cultured in the presence of the Rac inhibitor NCS23766 (Figure 4H–K). These findings suggest a likely role for Rac in regulating αSMA + myofibroblast differentiation in response to cataract surgery wounding.

### 3.5. Relationship between Rac and PI3K Signaling in Response to Cataract Surgery Wounding

**Figure 5:**
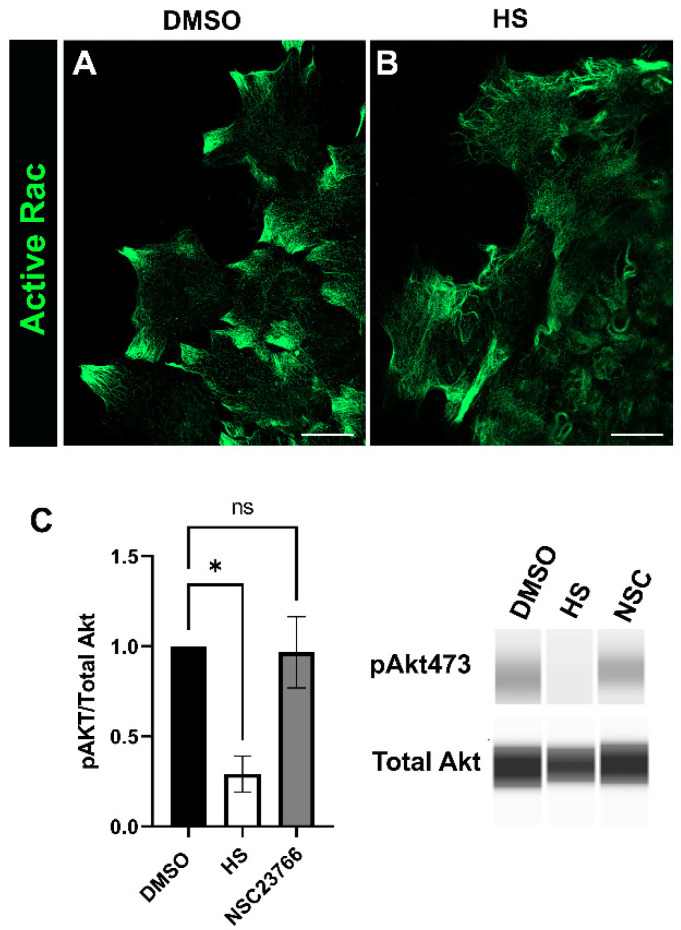
The link between Rac and PI3K signaling in promoting myofibroblast differentiation in response to injury. (**A**,**B**) Ex vivo post-cataract surgery explant cultures treated for 24 h from culture day 1 with either (**A**) the vehicle (DMSO) or (**B**) the p110α inhibitor HS-173 immunolabeled for active Rac and imaged at the leading edge by confocal microscopy. In control cultures active Rac localized to protrusions extended by leader cells and this distribution was altered by treatment with HS-173. Images are presented as a single optical plane from the collected confocal z-stack with each optical plane at 0.33 µm. Magnification bar = 20 µm. (**C**) Ex vivo post-cataract surgery explants were treated with vehicle (DMSO), the Rac specific inhibitor NSC23766 (NSC) or the p110α inhibitor HS-173 (HS) on day 1 through day 2 post-injury and subjected to immunoblot analysis for pAkt and total Akt. Quantification of the relative intensity of pAkt to total Akt normalized to DMSO is displayed in bar graphs (*n* = 3). Error bars represent S.E.M., * *p* ≤ 0.05, ns: not significant.

Our findings above revealed roles for both p110α and Rac signaling in promoting the transition of mesenchymal leader cells to αSMA+ myofibroblasts (Figure 2 and Figure 4). To determine the impact of inhibiting p110α on active Rac expressed by leader cells in the ex vivo cataract surgery cultures they were treated for 24 h with either vehicle or the p110α inhibitor HS-173 starting on day 1 post-injury. The cultures were immunolabeled for active Rac and imaged at the leading edge by confocal microscopy. Active Rac was present along filaments in the leader cells of both vehicle and HS treated cultures (Figure 5A,B), but with distinct patterns of organization. In vehicle controls, active Rac was most highly expressed in protrusions extended by the cells along the substrate (Figure 5A), as is also shown in the study in Figure 4. These active Rac-rich protrusive structures were absent in the leader cells of cataract surgery wounded explants cultured in the presence of the p110α inhibitor (Figure 5B). These findings suggest that p110α could impact Rac signaling by altering its localization within leader cells.

Rac is known to act both upstream and downstream of PI3K signaling [46]. Therefore, we also examined whether blocking Rac affected activation of the PI3K signaling pathway. For these studies, ex vivo post-cataract surgery cultures were treated for 24hrs with vehicle DMSO, the Rac inhibitor NSC2367 or the p110α inhibitor HS-173, starting on day 1 post-injury. Immunoblot analysis was performed to determine the activation (phosphorylation) of the PI3K downstream effector Akt (Figure 5C). While the p110α inhibitor HS-173 suppressed activation of Akt, inhibition of Rac signaling with NSC did not have a significant impact on PI3K signaling. Overall, our findings suggest that Rac signaling is not upstream of PI3K but rather may be regulated downstream of PI3K to signal myofibroblast differentiation.

### 3.6. Induction of Leader Cell Proliferation by PI3K p110α Is Linked to the Emergence and Persistence of Myofibroblasts Post-Mock Cataract Surgery Wounding

**Figure 6:**
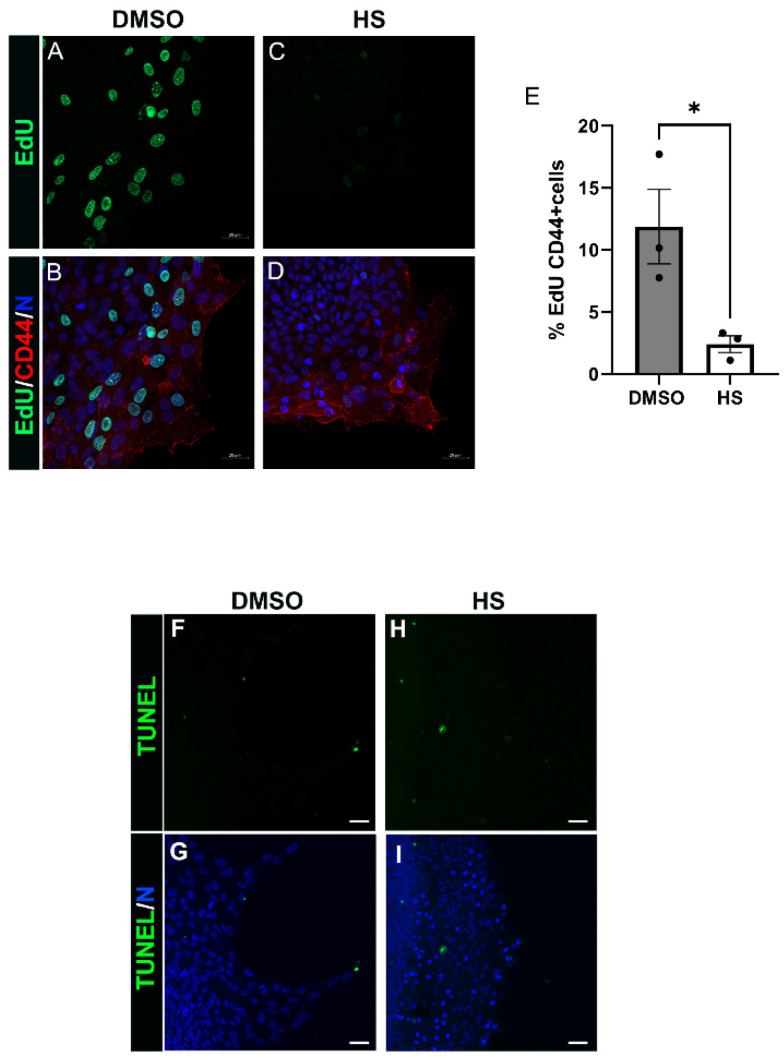
PI3K 110α signals CD44 leader cell proliferation. (**A**–**I**) Ex vivo post-cataract surgery cultures were treated with vehicle (DMSO) or HS-173 (HS) at day 1 for 24 h and assessed for proliferation by EdU (**A**–**E**) or apoptosis by TUNEL analysis (**F**–**I**). (**A**–**D**) Vehicle and HS-173 treated cultures were processed for EdU incorporation, immunolabeled for CD44 and co-stained for DAPI. (**E**) Quantification of the % CD44+ cells that were EdU+ for vehicle and HS treated ex vivo post-cataract surgery cultures are displayed in the bar graph. Inhibiting PI3Kα led to a significant decrease in leader cell incorporation of EdU, showing a requirement for PI3Kα in mediating CD44+ leader cell proliferation. (**F**–**I**) Vehicle and HS-173 treated ex vivo post-cataract surgery cultures were processed for TUNEL and co-stained for DAPI and imaged at the leading edge by confocal microscopy. Treatment with HS-173 did not cause any increase in TUNEL labeling of leader cells indicating that mesenchymal leader cell survival is not mediated by PI3Kα. Images are presented as a projection from the collected confocal z-stack with each optical plane at 0.49 µm (**A**–**D**) or 0.6 µm (**F**–**I**). Magnification bar = 20µm. Error bars represent S.E.M., * *p* ≤ 0.05.

PI3K signaling is a critical regulator of both cell proliferation and survival [23,52], both cellular processes closely linked to the pathological development of fibrosis in tissues [22,53]. To begin to elucidate how the PI3K p110α subunit promotes the fibrotic phenotype in response to lens wounding we investigated whether p110α regulates mesenchymal leader cell proliferation or survival. For these studies, ex vivo post-cataract surgery explants were treated for 24 h with vehicle or the p110α inhibitor HS-173 beginning at culture day 1. The impact of blocking p110α signaling on leader cell proliferation was determined by EdU incorporation (Figure 6A–E). The impact on cell survival was determined by TUNEL analysis (Figure 6F–I). EdU-labeled explant cultures were co-immunolabeled for CD44, a receptor expressed by the resident immune leader cell population post-wounding, and the cultures imaged by confocal microscopy (Figure 6A–D). The percent of CD44-labeled cells that were EdU positive was determined (Figure 6E), and the results showed that p110α signaling is critical to regulating CD44+ leader cell proliferation. In contrast, inhibition of p110α signaling had no effect on leader cell survival (Figure 6F–I). These findings reveal that PI3K p110α signaling plays a critical role in inducing proliferation of myofibroblast progenitor cells, a process essential to the establishment and expansion of myofibroblasts and the progression of fibrotic disease.

## 4. Discussion

There is a critical need to elucidate the pathobiological signaling mechanisms that drive the proliferation, differentiation, and persistence of the αSMA+/collagen I-producing myofibroblasts that are responsible for the progression of fibrotic disease. In this study we sought to understand the function of class I PI3K p110 isoforms activated in response to cataract surgery wounding in the integration of signaling inputs from the environment that induce myofibroblast emergence and persistence, leading to the development of fibrosis. This study reveals a requisite role for PI3K, specifically the PI3K p110α isoform, in signaling the mesenchymal resident immune leader cells that rapidly populate the wound edge to produce and organize a FN-EDA provisional matrix and their subsequent transition to collagen I producing αSMA+ myofibroblasts. Importantly, our studies show that injury-induced signaling through the PI3K p110α isoform is required for the proliferation, expansion, and persistent activation of this leader cell population, and the progression of fibrosis.

Our findings reveal that activation of the PI3K p110α isoform impacts many different aspects of the fibrotic process, each of which could be regulated through the engagement of distinct upstream receptor inputs and/or downstream signaling effectors. Our studies also demonstrate that other PI3K isoforms expressed by the lens, PI3K p110β or p110γ [36] were alone insufficient to signal the fibrotic response to cataract surgery wounding. The literature shows that PI3K p110α is recruited to the plasma membrane following activation of either RTKs or integrins, while the PI3K p110β and p110γ subunits are recruited to the membrane in response to GPCR signaling [54]. Therefore, our findings that p110α has an essential role in the induction of post-cataract surgery fibrosis suggest that RTK and integrin receptors are the predominant upstream inducers of this process. It is likely that these receptors function both independently and coordinately to regulate different effector pathways that are activated downstream of their activation of PI3K p110α. This outcome would explain the different aspects of fibrotic disease progression that we find are blocked by specific inhibition of p110α signaling. These features of fibrosis include the formation of a FN-EDA-rich matrix, the transition of resident immune cells at the leading edge of the wound to αSMA+ myofibroblasts, and the expansion of these myofibroblasts and their expression of collagen I. Studies have shown that FN fibrillogenesis, which is required to form an FN matrix, involves both the integrin effector FAK and the activation of PI3K [55]. We propose that coordinate signaling between integrins and the quintessential fibrotic regulator, TGFβ1 [14] is linked to the role of PI3K p110α signaling in the formation of a pro-fibrotic FN-EDA matrix microenvironment. Future studies are required to tease apart the distinct upstream regulators induced in response to cataract surgery wounding that is responsible for the activation of PI3K p110α and its many downstream targets that lead to a fibrotic response to wounding**.**

At the membrane, PI3K serves as a sensor of changes that occur in the extracellular environment, with the ability to integrate biochemical and mechanical cues to signal alteration in cell function [56]. Membrane bound PI3K converts phosphatidylinositol (4,5) biphosphate (PIP2) to the second messenger phosphatidylinositol (3,4,5) triphosphate (PIP3), which can engage and activate various downstream effectors, including Akt and PIP3 sensitive Rac-Guanine nucleotide exchange factors (GEFS) that displace bound GDP for GTP to activate Rac [45,46]. In this study, we discovered that Akt activation alone was insufficient to promote PI3K p110α induced myofibroblast emergence, evidence that other downstream effectors of PI3K p110α signaling are also involved. Our studies revealed that Rac activation also is required for the transition of leader cells to an αSMA+ myofibroblast phenotype post-mock cataract surgery. For these studies, we used two different Rac inhibitors, NSC23766, which inhibits Rac 1 binding and activation by the Rac specific GEFs Trio and Taim1 and EHop-016, which inhibits the association of active VAV2 with Rac 1. The Rac GEF, Vav is sensitive to PIP3 regulation [46,57], while Tiam localization to the plasma membrane appears to be independent of PIP3 [46,58]. There is also the possibility that other PIP3 regulated Rac GEFs, such as P-Rex1, are activated in response to lens injury to promote a fibrotic outcome. While we did not find a significant impact of inhibiting PI3Kα on Rac activity, our findings revealed a role for PI3K p110α in regulating the distribution of active Rac in leader cells. Future studies are required to further unravel the interplay of Rac and PI3K signaling in promoting fibrosis in response to cataract surgery. Lastly, it is important to point out that our studies do not exclude that Rac is being activated in a PI3K-independent manner.

It is well established that PI3K is a signaling intermediate in the induction of collagen synthesis [59,60,61,62,63,64], but less is known about the specific PI3K isoforms that regulate collagen production [65,66]. Our studies show that activation of PI3K p110α signaling is linked to the production of collagen I by the myofibroblasts that emerge post-cataract surgery wounding, providing a key element in the establishment of the fibrotic state and the tissue damage that occurs in fibrosis. A study by Hettiarachchi et al., showed that inhibition of the PI3K/mammalian target of rapamycin (mTOR) signaling axis was sufficient to block pulmonary fibrosis in an experimental bleomyocin mouse model [67]. This signaling pathway was linked to the expression of αSMA and hydroxyproline, and collagen deposition by the emergent myofibroblasts [67]. It will be of future interest to determine the repertoire of p110α downstream effector pathways, such as mTOR and the Akt-independently regulated Serum and glucocorticoid-inducible kinase 1 (SGK1), in the regulation of the emergence and persistence of myofibroblasts and their production of collagen I that is associated with the fibrotic wound repair outcome to cataract surgery wounding.

Importantly, we have linked PI3K p110α induced proliferation of activated lens resident immune cells at the wound edge to their transition to a myofibroblast phenotype and to the expansion of the myofibroblast population. Typically, cell proliferation is a prerequisite for differentiating cells to acquire a new phenotype [68,69]. Therefore, we propose that PI3K p110α induces the proliferation of CD44+ resident immune cells as a necessary step for the establishment of the new phenotype of myofibroblast. Once this phenotype is established, we propose that proliferation induced by PI3K p110α allows for the expansion and possible persistence of myofibroblasts. A role for the PI3K p110α isoform as a critical regulator of proliferation in the lens is supported by studies with the PI3K p110α lens conditional mouse KO mouse [70]. While our previous studies show that wounded lens epithelial cells, the follower cells in this wound-repair paradigm are induced to replicate within the first hour post-cataract surgery wounding [71], we now find that at a later time post-wounding replication is limited to the mesenchymal leader cell population.

Overall, our studies show an important link between activation of both PI3K p110α and Rac and the production of a pro-fibrotic matrix environment following a wounding, as well as the emergence and proliferation of myofibroblasts. These findings support the therapeutic targeting of PI3K p110α to treat lens fibrotic disease.
